# Fostering Motivation: Exploring the Impact of ICTs on the Learning of Students with Autism

**DOI:** 10.3390/children11010119

**Published:** 2024-01-18

**Authors:** José María Fernández-Batanero, Marta Montenegro-Rueda, José Fernández-Cerero, Eloy López-Meneses

**Affiliations:** 1 Department of Teaching and Educational Organization, University of Sevilla, 41013 Sevilla, Spain; batanero@us.es (J.M.F.-B.); mmontenegro1@us.es (M.M.-R.); jfcerero@us.es (J.F.-C.); 2 Department of Education and Social Psychology, Pablo de Olavide University, 41013 Sevilla, Spain

**Keywords:** ASD, ICT, student, motivation

## Abstract

Currently, the use of digital tools has led to significant changes in the educational system, favouring equity and the inclusion of students with educational needs. In this context, students with autism spectrum disorder (ASD) benefit from using these electronic devices to improve their learning experience. This study focuses on conducting a bibliometric analysis of the impact of information and communication technologies on the learning of students with ASD, with the aim of addressing two research questions. Through the analysis of three databases (Scopus, Dialnet, and Web of Science), a total of 24 articles related to the subject were collected. The results show that the use of different technological devices has numerous benefits for these students. Among the most prominent are the use of augmented reality and educational robotics, mainly providing improvements in academic performance, motivation and improved retention of knowledge acquired in the classroom. In conclusion, the clear need to train teachers in digital competencies and to intensify efforts in this line of research in order to improve the education of students, as well as to enrich the knowledge available to the scientific community, is highlighted.

## 1. Introduction

One of the most important issues in teaching–learning environments is to increase the quality of education for people with special educational needs and to facilitate their learning. In this context, developments in information and communication technologies (ICTs) in education have led to significant changes in the way educators can approach the learning of these students.

ICTs can be defined as innovations in telecommunications and computing (hardware or software) that enable the processing of information through devices that manage and organise this information [1]. In the last decade, the use of technology such as laptops, digital tablets or virtual platforms that are used at any educational level and as a resource to address student diversity has been increasing [2]. Therefore, the need to provide autonomy and inclusivity for students with disabilities in educational settings has become critical. ICTs play an essential role in providing the necessary support, ensuring that students with disabilities have opportunities comparable to those of their peers without limitations, enabling them to actively participate in society. 

In this sense, several initiatives have emerged in the field of ICT to provide support to overcome the difficulties faced by students with autism spectrum disorder (ASD). Following this line, ICTs represent a fundamental means of intervention, being a support that must be based on the essential conditions of an effective intervention programme [3]. This approach seeks to attend to the specific needs of each individual, complying with a series of conditions that include [4] fostering their physical and emotional well-being, promoting personal autonomy, facilitating the development of cognitive and communicative skills, as well as cultivating competencies for their effective interaction with other people and the environment. The numerous possibilities they provide for students with specific educational needs create the opportunity to acquire knowledge in a more practical way, guaranteeing equitable access to content, fostering autonomy and independence, and providing a more effective response to the demands of society. For this reason, it is essential to consider the development and implementation of technologies as a priority in the field of scientific research, with the aim of achieving sustainable and equitable progress for all students [5]. The effective management of these technologies is fundamental in education due to their capacity to offer opportunities to individuals and overcome possible impediments.

In this context, the integration of certain electronic devices in the classroom as an educational tool for students with ASD offers a suitable environment for the development of learning. Several studies support the benefits of ICTs for students, highlighting the importance of implementing learning through ICTs in familiar and school environments, thus facilitating the strengthening of their social skills [6]. Likewise, a review by Virnes et al. [7] confirmed that technology appears to be a highly motivating and appropriate medium for the learning and rehabilitation of skills that are often problematic for students with ASD. Furthermore, it should be added that the value of these tools lies in the fact that they allow for contextualised communicative situations and increase motivation regarding learning because they are different from what the learner is used to, adapting to the needs of each case [8].

Several studies have examined the particular difficulties faced by students with ASD in areas crucial to their academic and social growth. In line with this line of research, social interactions stand out as one of the main characteristics, encompassing the lack of development in skills such as joint referencing, the ability to share attention or common actions, the inappropriate use of non-verbal behaviours to regulate social interaction, and the presence of language and communication challenges [9].

This study seeks to fill a gap in the existing literature by focusing on an essential but underexplored aspect: the motivation of students with ASD through ICTs. Although the effectiveness and potential of ICTs in inclusive education has been recognised, current gaps in the research do not allow for an understanding of how these technologies impact students with ASD. This approach is vital, as motivation plays a central role in effective learning and skill development. Thus, the primary purpose of this systematic review is to examine the existing literature on how the use of ICTs influences the learning of students with ASD, with the aim of discerning their impact and the challenges related to their implementation. Through this approach, we aim to provide a more accurate and comprehensive view of how ICTs can positively influence motivation and skill development in this group of learners, thus benefiting not only their educational experience but also their active participation in society. In this context, the following research questions are explored:

RQ1. What are the current trends in research on the integration of ICTs to enhance the educational experience of students with autism spectrum disorder?

RQ2. What impact does the use of technology have on the learning of students with ASD?

## 2. Theoretical Framework

The integration of information and communication technologies (ICTs) in special education, especially in the context of autism spectrum disorder (ASD), represents a crucial advance in significantly improving students’ educational experiences [10]. These technological tools not only facilitate access to specialised educational resources, but also serve as powerful enablers to address specific challenges associated with students’ educational needs. The adaptability of ICTs makes it possible to personalise the teaching process, catering to the individual needs of each student, whether in terms of communication, social interaction or learning styles. The strategic implementation of ICTs provides a more inclusive educational environment, where technology becomes a fundamental ally to cultivate cognitive, social, and communicative skills. In this context, the integration of ICTs not only enriches the learning experience of students with ASD, but also enhances their active participation in society, contributing to the construction of an equitable and enriching educational environment [11].

ASD, a neurodevelopmental disorder, causes significant challenges in the communication, social interaction, and behaviour of those who experience it, affecting their daily lives and learning [12]. Against this backdrop, the increasing use of ICTs is revealed as a fundamental resource to address and improve the educational experiences of people with ASD.

In line with the growing trend, there has been a significant increase in the use of technologies to address the educational needs of students with ASD, which may be due to the numerous opportunities offered by technologies to improve teaching, adapting it to the specific characteristics of these students. This technological approach encompasses not only access to specialised educational resources, but also the development of tools and applications designed to facilitate communication, improve social skills, and provide a more inclusive educational environment [13].

Sanromà-Giménez et al. [14] argued that the use of technological devices with individuals with ASD can play a crucial role in providing a wide range of meaningful and motivating learning resources, situations, and scenarios. Therefore, an educational action can be implemented that offers individualised and personalised attention, adapted to the educational needs of the students, improving the teaching, and learning process according to their learning pace. Following this line, the use of ICTs has a positive impact on the teaching–learning process of students with ASD, as it favours a learning approach that takes into account and respects the pace, maturation and cognitive and motor level of each individual [15]. In this line, studies such as those by Aljehany and Bennet [16] argue that video applications are preferred by people diagnosed with autism. 

These digital tools play an essential role in promoting the learning of students with special educational needs by introducing new methodologies and didactic strategies, as well as facilitating communication and interaction, overcoming individual differences [17].

In this sense, other studies have investigated the improvement of communication skills in children with ASD through the implementation of ICTs in educational settings. Participating teachers play a crucial role in the procedure, and the results obtained focus on their work experience, the application of ICTs for students with ASD and the improvement of communication skills. Their study highlighted that the vast majority of participants implement ICT methodologies and observe significant improvements in communication skills, especially when supported by visual and audio tools. These results provide valuable insights into the positive impact of ICTs on the communicative development of children with ASD, supporting the importance of their integration in inclusive educational settings [18]. However, there is also support for the opportunities that effective ICT integration can provide as a valuable tool for improving literacy skills in students with ASD, opening up new perspectives in the implementation of inclusive and personalised approaches in education. Along these lines, they constitute both a tool and an effective means to address the difficulties encountered in working with students. These resources not only captured the attention of students with ASD, but also provide the opportunity to maximise the performance of the visual channel in students with ASD. They focus on the development of literacy skills through the use of pictograms in virtual environments [19].

The value provided by digital tools lies in their ability to facilitate contextualised communicative situations, which has a significant impact on increasing motivation for learning by presenting a different approach to what the student is used to. These tools are adapted to the specific needs of the individual [8]. However, it is important to stress that they should not be used in isolation. It is highly beneficial for students with ASD to combine these tools with diverse methodologies. This combination enables richer educational strategies, contributing to the optimal development of communication skills and social interaction [20].

However, it has been shown that the access that this group has to technological resources varies according to the environment in which they develop. In this regard, a recent bibliometric review study concluded that there are various factors that can become facilitators or barriers for this group to access these tools and/or make optimal use of them [21].

On the other hand, it is essential to consider that the effectiveness of ICT-based interventions for students with ASD can vary widely depending on the individual characteristics and specific needs of each student [22]. A crucial challenge we face in the successful implementation of these technologies in educational settings lies in the digital readiness of teaching staff. In order for ICTs to be used efficiently and meaningfully, teachers need to be trained and updated in the use of these tools and understand how to adapt them to the particular needs of students with ASD [23]. This involves not only acquiring technical skills, but also developing a deep understanding of how these technologies can support the learning and skill development of students with ASD [24].

## 3. Methods

The design of this study was adapted from the Joanna Briggs Institute (JBI) approach to conducting systematic reviews [25]. Also, to address the research questions defined in this systematic literature review, we identified and analysed peer-reviewed publications with a precise strategy following the criteria set out in the PRISMA statement [26]. Furthermore, the R program, Bibliometrix, and the VOSviewer 1.6.15 software were used to analyse this field of research [27].

### 3.1. Search Strategy

To collect data, three databases were searched, namely Web of Science, Dialnet and Scopus, from 2014 to 2023. The selection of these three databases was justified for three main reasons. Firstly, the prestige and international reputation of these platforms is considered, as they are recognised as the primary sources for accessing publications with the highest impact in the field. Secondly, in terms of the sample, their representativeness is supported by the international reputation of these databases and their strict compliance with indexing protocols. Third, the possibility that these databases are complementary is envisaged, even though there may be some overlap in their coverage and persistent biases in certain disciplines may be revealed, as indicated by Mongeon and Paul-Hus [28] in their work on citations. The underlying purpose of this review was to provide a comprehensive overview of research in this area.

In order to find research relevant to the subject area, specific search terms were used in both English and Spanish. These terms were applied in the fields of titles, abstracts and keywords to identify and compile research relevant to the specific area. To narrow the search, the following search terms were used: (“ASD” OR “TEA”) AND (“ICT” OR “TIC”) AND (“Motivation” OR “Motivación”) AND (“Autism” OR “Autismo”) AND (“Technology” OR “Tecnología”). To make the study more rigorous, the combined use of Boolean operators “AND” and “OR” was employed in the different searches. The search for articles was limited to the last decade (published between 2014 and 2023). The search strategies used in the systematic review are included in Table 1.

### 3.2. Inclusion and Exclusion Criteria

As a result of the initial search, 268 records were identified in the selected databases. Duplicate publications (n = 142) in the different databases were also eliminated. The remaining 126 records were assessed against the selected inclusion and exclusion criteria to ensure their relevance to the review (Table 1). To apply the inclusion and exclusion criteria, the PICoS strategy was used: Population, Phenomenon of Interest, Context, and Study Design [29] (Table 2). The population responded to the search delimitation criteria (temporal delimitation, type of document, language, area), the phenomenon of interest was based on extracting proposals directly related to the subject, the context investigated was education, and the study design prioritised quantitative and qualitative articles that analysed this subject. These studies were reviewed independently by two authors to ensure objectivity and minimise bias in study selection. 

A total of 101 studies were excluded because they did not meet the established inclusion and exclusion criteria (35 were not scientific articles published in a peer-reviewed journal, 31 were published before 2014, 23 were published in a language other than English or Spanish, and 12 did not analyse the impact of ICTs on the motivation of students with ASD in an educational setting).

### 3.3. Methodological Quality Assessment

Before studies were included in the systematic review, the methodological quality and risk of bias of each of the remaining 25 studies were critically assessed. For this purpose, the Joanna Briggs Institute (JBI) tool was used to analyse the methodological quality of the study and to determine the extent to which a study addressed the possibility of bias in its design [30].

The JBI instrument “Checklist for Diagnostic Test Accuracy Studies” was employed by two reviewers independent of the study, who undertook a critical evaluation of the data. Due to some disagreements between the reviewers, a third reviewer was requested to assess the data. To be included, studies had to reach a minimum quality threshold of 5 points. Most of the included studies were of high quality, so only one study was excluded in this review. The checklist used was designed according to the JBI methodology and included the assessment criteria presented in Table 3.

### 3.4. Data Selection and Extraction 

**Figure 1:**
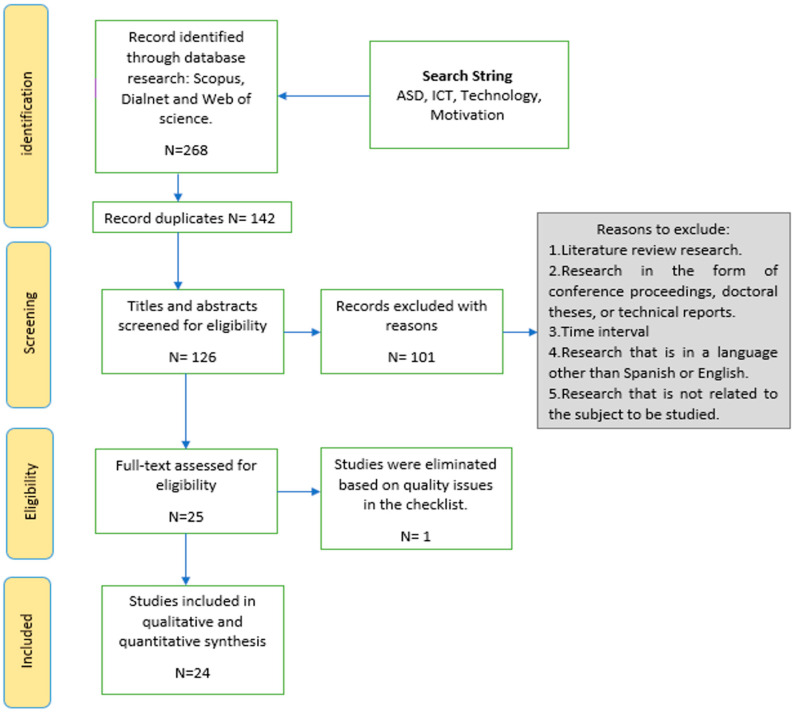
Flow chart of the selection process.

After assessing the methodological quality of the studies, a total of 24 studies were included in this systematic review. The flow chart of the study selection process, based on the Preferred Reporting Items for Systematic Reviews and Meta-Analyses (PRISMA) guidelines, is presented in Figure 1.

For the analysis of the systematic review data, a bibliometric analysis was carried out using R statistical programming, together with the “Bibliometrix” and “Biblioshiny” libraries. This open access library enables importing data from various indexes such as Scopus and Web of Science, among others [31]. On the other hand, the research problem was analysed on the basis of a semantic network generated through the VOSviewer 1.6.15 software [32]. In it, the most significant lines of research in this field of research were categorised. The scientific productions included in the systematic review are presented in detail in Table 4.

## 4. Results

After compiling the most significant studies related to the impact of the use of ICTs on students with ASD, the most outstanding results of scientific research are presented below.

**Figure 2:**
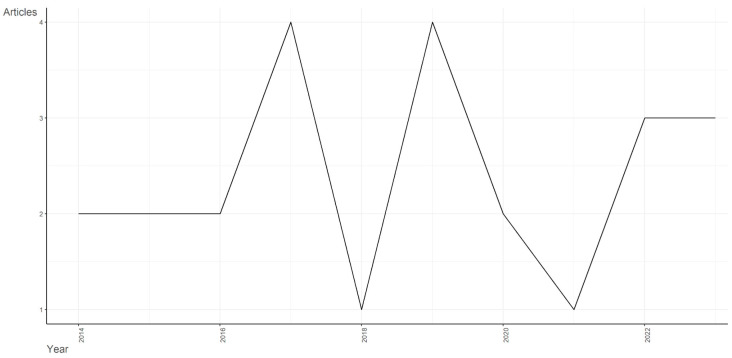
Annual scientific production.

In Figure 2, a graph illustrating the annual scientific production about the impact of ICTs on the learning of learners with autism can be seen. After an exhaustive process of collecting scientific articles, it is evident that the years 2017 and 2019 stand out as periods of greater scientific production in the literature on this topic. On the other hand, a decrease in the production of scientific literature is observed in 2018 and 2021. However, from 2022 onwards, there is an upward trend in research, reflecting a growing interest and relevance in the field of education.

**Figure 3:**
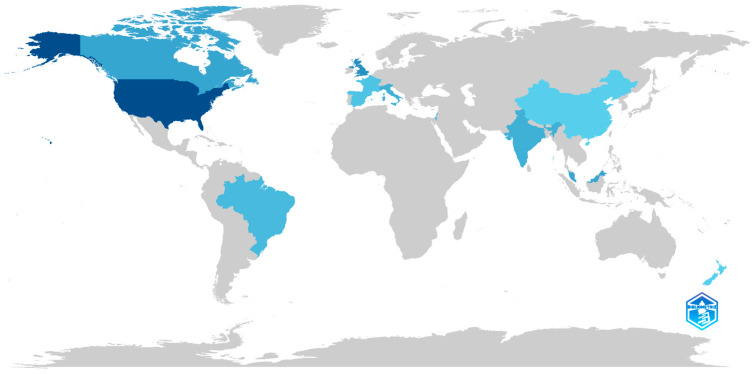
Scientific production by country.

Figure 3 shows a map reflecting the scientific production by country of origin on the subject from 2014 to 2023. The countries that have carried out research are shown in blue, with the darkest shades being those that have produced the most scientific output during the established time interval. In this sense, the United States is presented as the country with the highest production. Following this line, the United Kingdom is positioned as the country with the second highest number of publications, followed by Canada, Italy, India and Malaysia. However, other countries have also carried out research, but to a lesser extent, such as Brazil, Spain, France, China and New Zealand.

Along these lines, a composite map was created using the Bibliometrix programme to provide an overview of the main research topics. The location of a bubble on the map was interpreted considering both the density and centrality of the topic. The classification of the topics was achieved by applying four different groups, according to the concepts of centrality and density, as proposed by [56]:

- Driving Themes: These are characterised by being solidly developed areas in the field, being fundamental for the organisation of the topic of study. In the thematic map, these prominent themes are located in the upper right quadrant, highlighting their importance and centrality.
- Core Themes: These are of great relevance, although they have not yet reached full development in the research field. They are placed in the lower right quadrant, indicating their potential importance and the need for further development.
- Emerging or Declining Themes: These are characterised by low density and centrality, representing areas that are emerging or experiencing a decline in research. They are located in the lower left quadrant, indicating their position in the research spectrum.
- Niche Themes: These have high density but low centrality, being highly specialised and peripheral areas in the field of study. These unique themes are found in the upper left quadrant, indicating their specialisation and relative separation from the central core of the research.

In this sense, through the systematic literature review, it became evident that the topics of “assistive technologies”, “augmented reality” and “mental health” were among the driving themes. In terms of niche themes, only “motivation” was highlighted as a central theme. In the core themes, the terms “autism”, “technologies” and “collaboration” were highlighted. Finally, in the emerging or declining themes, the theme “ASD” was indicated.

**Figure 4:**
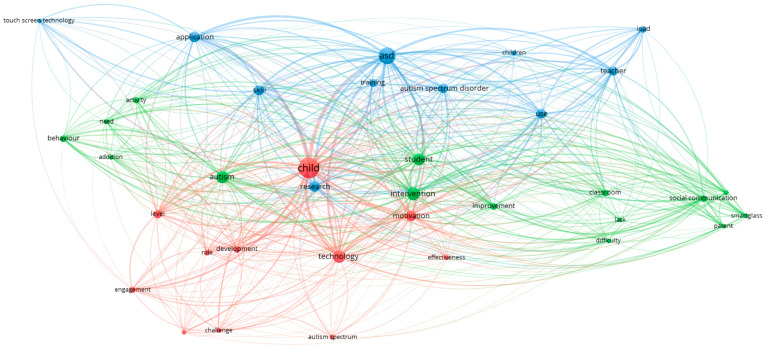
Keyword co-occurrence map.

VOSviewer was used with the 24 articles collected in the systematic review to create a semantic map by analysing the keywords in the articles. Figure 4 visualises the connections between the keywords through groupings of terms, also known as clusters. This figure allows us to visualise the relationships and connections between different studies, authors, themes or concepts within the literature reviewed. The graphical representation of networks facilitates the identification of patterns, thematic clusters and the understanding of the general structure of the field of study. In this way, it helps to identify emerging trends and gaps in research by analysing the frequency and evolution of key terms over time. This allows reviewers to identify areas where research is abundant and those where more attention is needed. In this sense, this tool allowed us to identify three key thematic clusters: 

Cluster 1: This cluster is identified in blue and represents the need for teacher training in the use of electronic devices with the aim of using them with students with ASD. Some of the descriptors include training, teacher or skill.

Cluster 2: Represented in green, this cluster is related to the barriers that students with ASD may encounter through the use of ICTs. Some of the most representative terms include difficulty, addiction, intervention.

Cluster 3: Represented in red, this cluster is closely linked to the impact of the effective use of technologies on learners with ASD. Some of the descriptors include development, effectiveness, motivation.

## 5. Discussion

The discussions are organised around the two research questions posed at the beginning of this research work.

**RQ1.**  *What are the current trends in research on the integration of ICTs to improve the educational experience of students with autism spectrum disorder?*

The scientific production about the use of ICTs to improve the educational experience of students with ASD shows that, despite the decrease in production between 2018 and 2021, there was a significant increase in the last year. This upturn could suggest an increase in awareness and importance attributed to this topic, driven by technological developments, changes in educational policies or a greater understanding of the effectiveness of ICTs in the context of ASD. 

If we examine the production by country of publication, the United States stands out as the country with the highest number of publications in this field, a finding that is consistent with previous research [57,58]. This phenomenon can be attributed to the fact that the most recent studies in the United States indicate that 1 in 59 individuals receive a diagnosis of ASD [59]. This has led to a significant increase in investment in research and the dissemination of information related to ASD. Geographical diversity in research highlights the global relevance of this topic, with evidence of its importance in various parts of the world such as Spain, Canada and the United Kingdom. A wide diversity of research sources will enrich the quality of the research and foster international collaboration in the search for effective solutions for the integration of technology in the education of individuals with ASD.

**RQ2.**  *What impact does the use of technology have on the learning of students with ASD?*

The analysis of the studies included in the systematic review established a positive relationship between the use of information and communication technologies (ICTs) and students with ASD. In other words, the findings of the reviewed studies suggest that the implementation of ICTs has a positive impact on the teaching–learning process of students with ASD, mainly by increasing students’ motivation.

ICTs provide an environment conducive to developing engaging and playful digital tools and applications, which can significantly influence the participation and interest of students with ASD in educational activities. The key to understanding this benefit lies in the ability of ICTs to adapt to the individual needs of students with autism. These tools can be customised to address the particular interests of each student, allowing them to establish a more meaningful connection with the content. This approach can be especially crucial for students with autism, as it can contribute to the reduction in anxiety and foster a more positive attitude towards learning [60,61]. Furthermore, scientific evidence supports the notion that the use of technology can be an effective instructional strategy to improve students’ skills [62]. Ultimately, given that the well-being and happiness of people with autism may depend on ICTs, there is a need to devote more effort to this area of research [63].

Likewise, the findings of this study reveal that the use of different devices has an impact on these students. Among them is the incorporation of augmented reality, the implementation of which has been proven to be a highly beneficial resource, especially in the context of students with ASD. Numerous studies have pointed out that the use of augmented reality not only enriches learning experiences, but also plays a key role in enhancing the motivation of students with autism, along with fostering a clear improvement in motivation when using this tool. The interactive and visually stimulating nature of augmented reality provides a more engaging and personalised learning environment, which can be particularly effective in maintaining the attention and interest of students with ASD [64]. In this sense, ICTs contribute to consolidating the learning process of students with specific educational support needs and, consequently, increase the number of opportunities to establish social relationships with other individuals, thus favouring their integration into the mainstream classroom environment thanks to student motivation [65].

The implementation of robotics as an educational resource resulted in significant improvements in students’ social and communication skills. Progress was also observed in cognitive and academic skills, including attention, concentration and problem solving, highlighting the ability of robotics to stimulate interest in learning. This study also indicated gains in motor skills and coordination, emphasising the positive impact of interaction with robots on physical development [66]. The potential of robotics as an effective tool to support inclusive learning and foster holistic development in the context of students with ASD is highlighted [50].

Along these lines, other studies reveal that the use of touchscreen devices such as iPads or mobile phones improves students’ motivation to learn and to communicate, as the applications installed on these devices arouse the interest and attention of these students [41]. The use of iPads is strongly associated with increased active participation, improvements in social communication skills and gains in language development. In addition, academic benefits were recorded, with improvements in attention, memory and problem solving. Along these lines, they also stood out for personalising learning to individual needs, promoting autonomy and independence in everyday tasks [43].

However, the use and implementation of game-based learning for students with ASD needs to be valued, as it has been shown to have enormous potential in terms of academic performance and motivation in the classroom. Several studies have indicated that those students who opted for the micro-worlds game-based system were able to decrease their cognitive load. In addition, they experienced significant improvements in motivational skills for learning, which facilitated the understanding of novel concepts. Furthermore, progress was observed in personality aspects related to the strengthening of interpersonal skills and self-efficacy. These results also demonstrated significant progress in learning achievement compared to the conventional method supported by educational technology [48].

In this sense, the findings of the systematic review show some challenges for the implementation of ICTs in the teaching and learning process of students with ASD. First of all, it is essential to provide training to teachers on the use of ICTs for diversity, in particular to serve students with ASD in order to ensure an inclusive and effective education. Educators play a crucial role in adapting and supporting the use of ICTs to meet the specific needs of students with autism. This process involves a thorough understanding of the available technological tools and the implementation of appropriate pedagogical strategies [24]. In this context, a clear concern has been expressed regarding the limited technological competencies that teachers possess in relation to the use of digital tools for the purpose of strengthening students’ learning and personal autonomy [67].

On the other hand, it is necessary to address more extensively the considerable challenge posed by the scarcity of technological resources to support the learning and development of students with autism in the current educational context. The lack of tools and technologies adapted to the specific needs of these students imposes significant barriers in their education. This limitation not only affects accessibility to pedagogical materials, but also impacts the implementation of teaching methodologies that could be highly beneficial for their cognitive, communicative, and social development [21].

For teachers and policy makers, considering these findings in the context of the diverse needs of ASD can be eye-opening. Exploring the practical implications and addressing challenges such as teacher training and the availability of adapted technological resources can significantly improve educational support for students with ASD. The authors are encouraged to explore these practical implications to enrich the application of these findings in real educational settings.

## 6. Conclusions

The impact of ICTs on the learning of students with autism is evident, underlining its positive influence on motivation and the development of both social and cognitive skills. The integration of ICTs creates a stimulating and adapted educational environment, fostering interest, active participation and improvement of the teaching and learning process of students with ASD. The personalisation of digital resources and the flexibility they provide allow individual needs to be addressed more effectively, thus promoting more meaningful learning. However, the notion of strategic use of ICTs should be considered not only to strengthen the intrinsic motivation of students with autism, but also to enhance their personal development skills in their daily lives. 

However, there are recognised challenges to be overcome when integrating technologies such as the technology gap and the need for adequate training of educational staff. The lack of digital skills supported by several studies is a critical aspect. Therefore, a continued focus on the effective integration of ICTs in inclusive educational settings can open up new opportunities to maximise the learning and development potential of students with autism. The combination of appropriate training and the careful implementation of technologies can create an enriching and accessible educational environment for all students.

### 6.1. Limitations 

Despite the thoroughness of this systematic review, we restricted our search to studies in English and Spanish, which may have excluded informative studies in other languages. Similarly, although three databases were used, it is possible that some relevant studies may have been omitted. 

### 6.2. Recommendations for Practitioners and Researchers

- Prioritise Digital Skills Training:

It is recommended to focus efforts on training teachers in digital competencies, ensuring that they are equipped to effectively integrate information and communication technologies (ICTs) in inclusive educational settings.

- Overcoming Technological Barriers:

It is recommended to actively address technological barriers, such as the digital divide, by ensuring equitable access to ICTs for all students with autism. This may include initiatives to provide appropriate devices and connectivity.

- Foster Interdisciplinary Collaborations:

Practitioners and researchers are encouraged to collaborate in an interdisciplinary manner, integrating expertise from special education, educational technology and psychology to optimise the implementation of ICTs in the education of students with autism. Interdisciplinary collaboration can enrich research by providing a holistic perspective on how ICTs impact the learning of students with autism.

## Figures and Tables

**Table 1:** Search strategy.

Nº	Search Strategy
#1	(“ASD” AND “ICT” AND “Motivation”) OR (“TEA” AND “TIC” AND “Motivación”)
#2	(“ASD” AND “Technology” AND “Motivation”) OR (“TEA” AND “Tecnología” AND “Motivación”)
#3	(“Autism” AND “ICT” AND “Motivation”) OR (“Autismo” AND “TIC” AND “Motivación”)
#3	(“Autism” AND “Technology” AND “Motivation”) OR (“Autismo” AND “Tecnología” AND “Motivación”)

**Table 2:** Inclusion and exclusion criteria.

PICoS Strategy	Inclusion Criteria	Exclusion Criteria
Population	Indexed in Web of Science, Dialnet or Scopus	Publication not indexed in these three databases
	Scientific article published in a peer-reviewed journal	Not an article published in a scientific journal
	Published between 2014 and 2023	Published before 2014
	Articles published in English or Spanish	Article published in a language other than English or Spanish
Phenomenon of interest	Analyses the impact of ICTs on the motivation of learners with ASD	Does not analyse the impact of ICTs on the motivation of students with ASD
Context	Focuses on the field of education	Does not focus on the field of education
Study design	Empirical research	Not empirical research

**Table 3:** Checklist.

Checklist	Yes (1 Point)	No (0 Point)
Is the aim of the research clearly specified?	✓	X
Is the participant sample students with ASD?	✓	X
Are the instruments used for data collection appropriate?	✓	X
Are the results obtained useful for the scientific community?	✓	X
Are the authors’ conclusions based on the data analysed?	✓	X
Are recommendations made for future research?	✓	X
Are the limitations of the research included?	✓	X

**Table 4:** Articles included in the systematic review.

Author	Year	Methodology	Results
McEwen [33]	2014	Quantitative	The use of tactile technologies (iPod) favours communication, motivation, attention of students with ASD
Stockall and Dennis [34]	2014	Qualitative	The use of technology promotes motivation and empowerment of students with ASD.
Hochhauser et al. [35]	2015	Qualitative	The application of video modelling supports the competence and intrinsic motivation of students with ASD.
Parsons [36]	2015	Quantitative	A virtual reality game can improve the skills and motivation of these students.
Kamaruzaman et al. [37]	2016	Qualitative	Touchscreen technology enhances learning for students with ASD
Rani et al. [38]	2016	Qualitative	Mobile technology is beneficial for motivation and participation of students with ASD
Brodhead et al. [39]	2017	Qualitative	Video-based technology provides benefits for students with ASD
Espósito et al. [40]	2017	Qualitative	The use of tablet applications increases the cognitive and social skills of students with autism.
Sankardas and Rajanahally [41]	2017	Qualitative	The use of electronic devices (iPad) in the classroom promotes motivation and communication of students with ASD.
Sahin et al. [42]	2018	Quantitative	Assistive technologies show improvement in communication and motivation of students with ASD
Eden et al. [43]	2019	Quantitative	The iPad is a useful tool to promote the teaching of students with ASD, but training is lacking.
Tuedor et al. [44]	2019	Quantitative	Reading support software supports the participation and motivation of students with ASD.
Worthington [45]	2019	Quantitative	Technologies favour students with autism
Wright et al. [46]	2019	Quantitative	Three-dimensional technology favours learning and motivation of students with autism, but there is a lack of training.
Faria et al. [47]	2020	Quantitative	AI-based interactive games promote concentration and motivation.
Khamparia et al. [48]	2020	Qualitative	The use of interactive learning environments enhances the learning process and motivation of students.
Mosher and Carreon [49]	2021	Qualitative	The use of augmented reality has potential to improve the social skills and motivation of students with ASD.
Laurie et al. [50]	2022	Qualitative	Robotic toys create different opportunities for autistic learners
Lledó et al. [51]	2022	Quantitative	The use of augmented reality has the potential to enhance the development of activities and motivation of students with ASD.
Wu et al. [52]	2022	Qualitative	Augmented reality favours social interaction and motivation of children with ASD
Gevarter et al. [53]	2023	Qualitative	The use of augmentative alternative communication through games favours the development of skills.
Mazon et al. [54]	2023	Qualitative	Intelligent tutoring improves mathematical skills and motivation of students with ASD
McGuinty et al. [55]	2023	Qualitative	Virtual and interactive technology promotes emotional regulation of students with ASD

## Data Availability

Dataset available on request from the authors.
